# The awareness of women on prostate cancer: a mixed-methods systematic review protocol

**DOI:** 10.1186/s13643-020-01513-4

**Published:** 2020-11-03

**Authors:** Ebenezer Wiafe, Kofi Boamah Mensah, Adwoa Bemah Boamah Mensah, Varsha Bangalee, Frasia Oosthuizen

**Affiliations:** 1 University of KwaZulu-Natal, Durban, South Africa; 2 Kwame Nkrumah University of Science and Technology, Kumasi, Ghana

## Abstract

**Background:**

Prostate cancer accounts for about 10% of cancers affecting and claiming the lives of men. Studies have reported that women are better than men in recognition of the early manifestations of various cancers. Besides, women have been recognized to show a profound interest in their partners’ health and hence, make observations that men do not know. Several studies have reported on the knowledge gaps of prostate cancer among patients and the general population. It is vital to comprehensively review the available evidence and identify research gaps in our current understanding of knowledge of women on prostate cancer.

**Methods:**

A search of bibliographic databases, MEDLINE (EBSCOhost), CINAHL (EBSCOhost), PsycINFO (EBSCOhost), Web of Science, and EMBASE (Ovid) will be undertaken from January 1999 to December 2019. The search will be limited to studies published in the English language. Duplication of studies will be removed using the EndNote citation manager. After deduplication, citations will be screened independently by two authors according to prespecified criteria. Data extraction and quality assessment of the selected studies will be done independently by two authors. Meta-analytic methods will be used where appropriate. The convergent segregated method of synthesis will be adopted in this review.

**Ethics and dissemination:**

Primary data collection will not be involved in this study, hence formal ethical clearance will not be needed. The results of the study will be presented through a peer-reviewed journal and conference presentation.

**Patient and public involvement:**

Patients or the public will not be engaged in the conduct of this study.

**Trial registration:**

Open Science Framework (OSF) registration DOI: 10.17605/OSF.IO/EYHF2

**Supplementary Information:**

The online version contains supplementary material available at 10.1186/s13643-020-01513-4.

## Background

Prostate cancer is a common neoplasm in men, and its occurrence keeps rising in many countries [1]. It is number 3 on the global list of the most common cancers that claim the lives of men [2, 3]. The incidence rate, mortality rate, and the rate of diagnosis of prostate cancer have been documented to be 0.5 million per year, a man per 2 min, and almost 2000 men per day, respectively [2, 4]. In addition to the global burden of the disease, prostate cancer accounts for about 10% of cancers affecting men [2]. Thus, prostate cancer is of significant public health interest.

A study conducted by Quinn and Babb indicated a reduction in prostate cancer mortality from early detection [2]. Other studies have also indicated the importance of the early detection of the disease to reduce mortality [5, 6]. In North America, the establishment of Rapid Access Prostate Clinic (RAPC) and more extensive advocacy of prostate cancer to the general population, with global campaigns such as Movember (i.e., movember.com), has decreased the mortality of the disease [7]. Hence, measures to improve the early detection of the disease among the general population can have a significant impact on the disease.

There are scientific shreds of evidence to support the claim that women can be trusted with family health [5, 8]. Blanchard et al. reported that women are better than men in the following aspects of cancers; the recognition of the early manifestations of various cancers, the timely perception of the barriers to seeking help, and the timely report of the identified barriers [5]. Women have reported on breast cancer and cervical cancer, where education has equipped them to be in control of their health [9]. Besides, women have also been recognized as individuals who show a profound interest in their partners’ health. Hence, they can make observations that men themselves do not observe about their health [9]. Much evidence exists on the critical role of women in decision-making in the treatment of their spouse or partner diagnosed with prostate cancer [10, 11]. Similar studies have also explored their potential role in screening the disease. Scant data exist on the knowledge of women on prostate cancer. Therefore, it is essential to map up evidence to determine women’s knowledge of prostate cancer as a public health means of reducing the disease.

A survey conducted by Fitzpatrick et al. concluded that the public and patients are aware of prostate cancer but with certain knowledge gaps. It concluded that measures have to be taken to address the identified knowledge gaps [4]. It is essential to gather information on the current knowledge of prostate cancer in the general population. However, this review focuses on women, as public health care largely depends on women’s contributions, particularly in health education [12]. The study aims to map up existing evidence about the awareness of women on prostate cancer.

### Review question

Do women have adequate knowledge about prostate cancer?

## Objectives of the study

1. To assess the knowledge-base of women on the signs and symptoms of prostate cancer.
2. To determine the awareness of women on the risk factors and causes of prostate cancer.
3. To ascertain the knowledge of women on the availability of screening guides for the early detection of prostate cancer.

## Methods and analysis

The mixed-methods approach will be employed in this systematic review. The Joanna Briggs Institute (JBI) reviewer’s manual, with a focus on mixed-methods systematic reviews, will be utilized [13]. The mixed-methods approach to research, which is among the first three widely used research approaches, has gained popularity due to its ability to analyze data obtained from quantitative, qualitative, and primary mixed-methods research papers [14, 15]. The mixed-methods approach is also appropriate to permit data triangulation to enhance the study validity [16]. In addition to this benefit, the use of mixed-methods has proven to be useful in comparing and contrasting quantitative and qualitative research findings. This results in either the quantitative findings supporting the qualitative findings or vice versa [16]. To ensure that findings that have the highest recognizable strength are obtained at the end of the systematic review, the role of the mixed-methods approach in this review is important [13]. This critical appraisal and synthesis is registered with Open Science Framework and can be accessed through 10.17605/OSF.IO/EYHF2 and will commence in July 2020.

### Eligibility criteria

#### Population of interest

The population of interest is women who are 18 years of age or above. The review will include research papers that involve women who are 18 years of age or above as part of the study population. In addition to the description of the interest population (women who are 18 years and above), the features of women in included studies will not be restricted to cultural/sub-cultural backgrounds and geographical locations.

#### Interest phenomena

The phenomena of interest for this review is the awareness of women on prostate cancer. Therefore, this review will involve studies that evaluated the knowledge of women aged 18 years and above on prostate cancer.

#### Context of interest

The context of interest will include but not limited to all studies conducted in women of all cultural/sub-cultural backgrounds and geographical locations.

#### Outcome

The review will consider research papers that have the knowledge of women on the signs and symptoms, causes and risk factors, and screening recommendations of prostate cancer as the outcome measures. The signs and symptoms of prostate cancer that would be of interest in included studies would not be limited to difficulty in passing urine, dysuria, and the need to frequently pass urine. Also, women’s ability to determine the asymptomatic nature of the disease would be taken into account. Recruited studies would be expected to report on increased age, family history of the disease, African decency, male gender, exposure to radiation, and cigarette smoking as part of the non-exhaustive list of risk factors and causes of prostate cancer.

### Types of studies to be included

This critical appraisal and synthesis would involve primary research papers that are qualitative, quantitative, and mixed-methods in nature. Focused group discussions (FGD), observation of study subjects (follow-up studies), in-depth interviews, and other forms of interviews will constitute the qualitative studies to be included in this systematic review without ignoring other studies that meet the criteria of qualitative studies. Quantitative studies will constitute descriptive studies, descriptive cross-sectional studies, and other studies that meet the requirements of quantitative studies. Studies that combined qualitative and quantitative designs will constitute the mixed-methods arm of this study on a condition that the qualitative and quantitative data can be explicitly extracted. This review will also include studies that have been published in the English language. To be able to map up current evidence, studies dating January 1999–December 2019 will be included in this review.

### Exclusion criteria

The following will be grounds for excluding papers for this review:

- Studies that were published before January 1999 or after December 2019.
- Studies that were not published in the English language.
- Studies that include women below the age of 18 years.
- Studies in which the age of included women cannot be established.
- Studies that did not indicate the number/percentage of included women.
- Studies that exclusively included male without any female gender (18 years and above).
- Studies conducted amongst women who were previously given education on prostate cancer.
- Studies that exclusively involved lesbian, gay, bisexual, transsexual/transgender, and queer/questioning (LGBTQ) participants.
- Studies that exclusively included healthcare professionals.
- Studies that exclusively involved healthcare and college/university students.
- Studies that do not include the outcome of interest.
- Book chapters.
- Reviews and overviews.
- Abstracts and conference papers.
- Dissertations and thesis.
- Commentaries and letters to editors.
- Studies published without abstracts.

### Information sources and search strategy

A comprehensive search strategy will be developed by the primary author (EW) to identify various publications related to the study. The following databases will be searched; MEDLINE (EBSCOhost), CINAHL (EBSCOhost), PsycINFO (EBSCOhost), Web of Science, and EMBASE (Ovid). The searched period will be from January 1999 to December 2019.

A triple stage approach to searching published literature will be adopted in developing the preliminary search strategy [13]. Firstly, MEDLINE (EBSCOhost) will be searched for articles relevant to this review. Subject terms (identified from the title and abstract of the relevant papers) and free text terms (identified from the description of the relevant papers) will be employed in the development of the preliminary search strategy. The details of the search terms can be found as a supplementary document at; 10.17605/OSF.IO/EYHF2. The preliminary search strategy, using MEDLINE (EBSCOhost), has been affixed (Additional file 1: Appendix 1). Secondly, the preliminary search strategy will be adopted in the search for relevant studies from CINAHL (EBSCOhost), PsycINFO (EBSCOhost), Web of Science, and EMBASE (Ovid), respectively. The citation list of the selected studies for the review will be scanned for additional studies. Search findings, which will be in the English language, will be compiled independently by two authors (EW and KBM).

The total search findings of EW and KBM will be compiled and duplicate papers, removed with the help of the EndNote X8 software, to generate a final list of search findings for the review.

### Screening and selection of studies

The final list of search findings will be independently screened by two reviewers (EW and KBM). The titles and abstracts of the selected studies will initially be screened for inclusion and exclusion in the review. Studies that are selected after the screening of the titles and abstract will undergo full-text reading by the same independent reviewers. The selection for data extraction will be done by a careful comparison of papers that successfully passed the screening stage to the established inclusion and exclusion criteria. Search findings that present disagreements between the independent reviewers (EW and KBM), during the screening and selection stages, will be resolved through discussions with the third reviewer (ABBM). Kappa coefficient of 0.6 or more would be used to establish an agreement between the two independent reviewers (EW and KBM) during the screening and selection phase [17]. Reference management will be done by EW.

### Data extraction

The JBI quantitative and qualitative data extraction tools, Additional file 2: Appendix 2 [18–20] will be adopted to extract data from search findings. The convergent segregated approach to data synthesis and data integration recommends using separate data extraction tools for this review [13]. Data that will be extracted from the included studies will not be restricted to the study title, principal investigator (lead author), year of publication, the country in which the study was conducted, the study sample size, the study design, ethnic background of study participants, key discoveries of the study, the limitations of the study, and conclusions made by authors. Two reviewers (EW and KBM) will independently extract both quantitative and qualitative data. The outcome of the individual data extracted by these two authors will be merged to generate a final pool of data for the review. To account for missing data, EW will contact the authors of the primary studies through electronic mail. In an instance where authors of primary studies do not respond to electronic mails sent, the reviewers would consider it as “missing data” without making any assumptions. Disagreements that may arise when building the final pool of data will be resolved through discussions with ABBM.

### Assessment of methodological quality

The quality assessment of the review will adopt and adapt a quality appraisal tool employed in a review conducted by Mensah et al., Additional file 3: Appendix 3 [21]. The tool has 5 general quality assessment criteria and 3 specific quality assessment criteria to be scored over 100%. The design of the tool will permit the comparison of scores obtained by individual literature to the scoring benchmark of weak (0–33.9%), moderate (34–66.9%), and strong (67–100%) [21]. This quality appraisal stage will be done independently by two authors (EW and KBM). It is recommended that a minimum of two reviewers be involved in the appraisal of the quality of studies to be considered for data extraction and subsequent synthesis [20]. Hence, the need for EW and KBM to independently conduct a quality assessment of search findings that passed the screening and selection stage of the review. The results obtained from the quality assessment stage will be employed in determining the final inclusion or exclusion of a research paper in the review. Studies with weak methodological quality scores will be excluded. Disagreements arising from the results of EW and KBM will be addressed through discussions with ABBM.

### Data synthesis and integration

The convergent segregated approach will be employed in data synthesis and integration [13]. Quantitative data and qualitative data will be synthesized separately. The evidence that will be generated from the separate synthesis of quantitative and qualitative data will be integrated [13].

### Quantitative and qualitative data synthesis

The synthesis of quantitative data will be done through a narrative synthesis [13]. This is because the review has no interventional arm and is aimed at investigating the awareness of women about prostate cancer. Hence, a narrative synthesis would be performed on findings that would be extracted from included studies. It is imperative to note that random or fixed effects would not be employed in data synthesis. Also, subgroup or meta-regression analysis would not be performed.

The findings from the pool of qualitative data will undergo meta-synthesis through findings assembly and categorization based on shared meanings [22]. These categorized findings will undergo further analysis in an attempt to generate a wealth of evidence that will be easy to comprehend and will also be a true reflection of the awareness of women on prostate cancer concerning the underpinned review objectives [13].

In the event of the inability to perform a textual pooling, a narrative will be generated [22]. The synthesis by narrative will be performed according to the following approach; the findings obtained from the selected studies will undergo preliminary synthesis, the compiled data will be explored for linkages and the synthesizing process will be assessed for robustness [23].

### Integration of quantitative evidence and qualitative evidence

The evidence from the individual analysis of quantitative and qualitative data will undergo configuration [13]. The inability of evidence configuration will result in the narrative presentation of evidence [13].

## Discussion

This protocol is a blueprint to be followed for conducting a mixed-methods systematic review to map up evidence on the awareness of women on prostate cancer. To cover a wide range of primary literature, the protocol will employ the mixed-methods approach according to the JBI guidelines. By publishing the study protocol, we strengthen the clarity of the search strategy and reduce the risk of bias, particularly selective outcome reporting.

The wealth of evidence obtained from this systematic review will inform and support the involvement of women as health promoters and educators in the early detection and prevention of prostate cancer. The evidence will also be useful in improving the quality of life and survival rate of men living with prostate cancer.

### The potential limitations of this research

The study is confronted with selection bias resulting from the following:

1. The restriction of the literature search to the range of January 1999 to December 2019.
2. The consideration of studies published only in the English language for inclusion.
3. Limiting the literature search to five databases.
4. The exclusion of studies conducted in women who have received prior education on prostate cancer, exclusion of healthcare professionals, healthcare students, and college/university students.
5. The exclusion of studies that involved lesbian, gay, bisexual, transsexual/transgender, and queer/questioning (LGBTQ) participants.

## Supplementary Information

**Additional file 1:.** Proposed search strategy using Medline via EBSCOhost

**Additional file 2:.** JBI data extraction tools

**Additional file 3:.** Quality assessment tool

## Abbreviations

FDG: Focused group discussions
JBI: Joanna Briggs Institute
LGBTQ: Lesbian, Gay, Bisexual, Transsexual/transgender, and Queer/questioning
RAPC: Rapid Access Prostate Clinic
