# Supplementary material for: The awareness of women on prostate cancer: a mixed-methods systematic review protocol
Source: Syst Rev. 2020 Nov 3;9:253. doi: 10.1186/s13643-020-01513-4 (PMC7641856; doi:10.1186/s13643-020-01513-4)
Supplement: Supplementary file 1 — Additional file 1:. Proposed search strategy using Medline via EBSCOhost [file 13643_2020_1513_MOESM1_ESM.docx]

**APPENDIX 1**

**Proposed search strategy using Medline via EBSCOhost**

1. Awareness* OR Aware* OR Awarenesses*
2. Knowledge* OR Know* OR Knowledges* OR Knowledgeable* OR Known* OR Knowns* OR Know-how* OR Knowledge-base*
3. Signs and symptoms* OR Early signs and symptoms* OR Signs* OR Symptoms* OR Early manifestations* OR Manifestations* OR Symptomatology* OR Symptomatologies*
4. Risk factors* OR Risk factor* OR Causes* OR Cause* OR Predisposing factors* OR Predisposing factor* OR Triggering factors* OR Triggering factor* OR Trigger* OR Triggers* Underlying cause* OR Underlying causes*
5. Screening recommendations* OR Screening Recommendation* OR Screening Guideline* OR Screening Guidelines* OR Screening Guideline Recommendations* OR Screening Suggestions* OR Screening Procedures* OR Screening Methods* OR Diagnostic Guidelines* OR Diagnostic Methods* OR Diagnostic Tests* OR Diagnostic Tools* OR Diagnostic Recommendations* OR Diagnostic Procedures* OR Diagnostic Processes* OR Investigations* OR Laboratory Investigations* OR Clinical Investigations* OR Examinations* OR Laboratory Examinations* OR Clinical Examinations* OR Assessment Methods* OR Testing Tools* OR Testing Methods* OR Testing Guidelines*
6. Prostate cancer* OR Prostate malignancy* OR Prostate tumor* OR Prostate tumour*OR Prostate neoplasm*
7. Women* OR Woman* OR Ladies* OR Lady* OR Female* OR Females* OR Young women* OR Young woman* OR Young Lady* OR Young ladies* OR Wife* OR Wives* OR Mother* OR Mothers* OR Feminine* OR Maiden* OR Maidens OR Bride* OR Brides* OR Dame* OR Dames*
8. #1 OR #2
9. #3 OR #4 OR #5
10. #8 AND #9
11. #10 AND #6
12. #11 AND #7
