# Supplementary material for: The awareness of women on prostate cancer: a mixed-methods systematic review protocol
Source: Syst Rev. 2020 Nov 3;9:253. doi: 10.1186/s13643-020-01513-4 (PMC7641856; doi:10.1186/s13643-020-01513-4)
Supplement: Supplementary file 2 — Additional file 2:. JBI data extraction tools [file 13643_2020_1513_MOESM2_ESM.docx]

**APPENDIX 2**

**JBI DATA EXTRACTION TOOLS**

1. **JBI MASTARI DATA EXTRACTION TOOL FOR QUANTITATIVE RESEARCH**

Reviewer: ………………………………….. Date: …………………………………………..

Author: ……………………………………. Year: …………………………………………..

Journal: …………………………………… Record number: ……………………………….

**Study Method**

RCT Quasi-RCT Longitudinal

Retrospective Observational Other …….………………………..

**Participants**

Setting …………………………………………………….

Population ………………………………………………...

Sample size

Intervention 1…………….. Intervention 2 ………………. Intervention 3 ………………

**Interventions**

Intervention 1 ………………………………………………………………………………………

Intervention 2 …………………………………………..…………………………………………..

Intervention 3 …………………………………………………….………………...………………

**Outcome measures**

| **Outcome Description** | **Scale/Measure** |
| --- | --- |
|  |  |
|  |  |
|  |  |
|  |  |
|  |  |
|  |  |

**Results**

Dichotomous Data

| **Outcome** | **Control group number/total number** | **Treatment group number/total number** |
| --- | --- | --- |
|  |  |  |
|  |  |  |

Continuous Data

| **Outcome** | **Control group mean & SD (number)** | **Control group mean & SD (number)** |
| --- | --- | --- |
|  |  |  |
|  |  |  |
|  |  |  |

**Authors Conclusions**

**………………………………………………………………………………………………………………………………………………………………………………………………………………………………………………………………………………………………………………………………………………………………………………………………………………………………………………………………………………………………………………………………………**

**Comments**

**………………………………………………………………………………………………………………………………………………………………………………………………………………………………………………………………………………………………………………………………………………………………………………………………………………………………………………………………………………………………………………………………………**

Extraction of findings complete Yes No

**B. JBI QARI DATA EXTRACTION TOOL FOR QUALITATIVE RESEARCH**

Reviewer: ………………………………….. Date: …………………………………………..

Author: ……………………………………. Year: …………………………………………..

Journal: …………………………………… Record number: ……………………………….

**Study Description**

Methodology: ………………………………………….

Phenomena of Interest: …………………………….

Setting: ………………………………….

Geographical location: ………………………………..

Cultural background: ………………………………….

Participants: …………………………………………

Data Analysis: ……………………………………

Authors’ Conclusions: ……………………………………….

Comments: ……………………………………………..

Complete Yes No

| **Findings** | **Illustration form Publication**  **(Page number)** |  | **Evidence** |  |
| --- | --- | --- | --- | --- |
|  |  | **Unequivocal** | **Credible** | **Unsupported** |
|  |  |  |  |  |
|  |  |  |  |  |
|  |  |  |  |  |
|  |  |  |  |  |
|  |  |  |  |  |
|  |  |  |  |  |

Extraction of findings complete Yes No
