# Supplementary material for: The awareness of women on prostate cancer: a mixed-methods systematic review protocol
Source: Syst Rev. 2020 Nov 3;9:253. doi: 10.1186/s13643-020-01513-4 (PMC7641856; doi:10.1186/s13643-020-01513-4)
Supplement: Supplementary file 3 — Additional file 3:. Quality assessment tool [file 13643_2020_1513_MOESM3_ESM.docx]

**APPENDIX 3**

**QUALITY ASSESSMENT TOOL**

| SN OF SELECTED STUDY | GENERAL QUALITY ASSESSMENT CRITERA (GQAC) | | | | | SPECIFIC QAC | | | PERCENTAGE SCORE |
| --- | --- | --- | --- | --- | --- | --- | --- | --- | --- |
|  | A | B | C | D | E | F | G | H |  |
|  |  |  |  |  |  |  |  |  |  |
|  |  |  |  |  |  |  |  |  |  |
|  |  |  |  |  |  |  |  |  |  |
|  |  |  |  |  |  |  |  |  |  |
|  |  |  |  |  |  |  |  |  |  |

**KEY**

SN= Serial Number

GQAC= General Quality Assessment Criteria

QAC= Quality Assessment Criteria

A= Was the study population adequately represented by the selected study sample size?; B= Did the study document a response rate?; C= Did the data extraction instrument undergo a reliability assessment?, D= Did the data extraction instrument undergo a validity assessment?; E= Does this study contribute to primary source of data?; F= Was the knowledge of women on the signs and symptoms of prostate cancer assessed?; G= Was the knowledge of women on the causes and risk factors of prostate cancer determined? H= Was the knowledge of women on the availability of screening guides for prostate cancer detection ascertained?

**SCORING SCHEME**

Yes (Y)= 1; No (N) or Unclear (U) or Not Reported (NR)= 0

PERCENTAGE SCORE= 100((Selected Study Total Scores) ÷ (Sum of Assessment Criteria Scores))

INTERPRETATION OF SCORES: Weak= 0 – 33.9%, Moderate= 34% – 66.9%, Strong= 67% – 100%
